# Swimming training potentiates the recovery of femoral neck strength in young diabetic rats under insulin therapy

**DOI:** 10.6061/clinics/2019/e829

**Published:** 2019-04-19

**Authors:** Gilton de Jesus Gomes, Ricardo Junqueira Del Carlo, Márcia Ferreira da Silva, Daise Nunes Queiroz da Cunha, Edson da Silva, Karina Ana da Silva, Miguel Araújo Carneiro-Junior, Thales Nicolau Prímola-Gomes, Antônio José Natali

**Affiliations:** I Departamento de Educacao Fisica, Universidade Federal de Vicosa, Vicosa, MG, BR; II Departamento de Veterinaria, Universidade Federal de Vicosa, Vicosa, MG, BR; III Instituto Federal de Minas Gerais, Sao Joao Evangelista, MG, BR; IV Universidade Federal dos Vales do Jequitinhonha e Mucuri, Diamantina, MG, BR

**Keywords:** Diabetes, Exercise Training, Bone Fragility, Collagen Fibers

## Abstract

**OBJECTIVE:**

To test whether swimming training benefits femoral neck strength in young diabetic rats under insulin therapy.

**METHODS:**

A total of 60 male Wistar rats (age: 40 days) were divided equally into the following six groups: control sedentary, control exercise, diabetic sedentary, diabetic exercise, diabetic sedentary plus insulin and diabetic exercise plus insulin. Diabetes was induced with a unique intraperitoneal injection (60 mg/kg body weight) of streptozotocin. Seven days after the injection and after 12 hours of fasting, the animals with blood glucose levels ≥300 mg/dL were considered diabetic. Seven days after the induction of diabetes, the animals in the exercise groups were subjected to progressive swimming training (final week: 90 min/day; 5 days/week; 5% load) for eight weeks. The animals in the insulin groups received a daily dose of insulin (2-4 U/day) for the same period.

**RESULTS:**

Severe streptozotocin-induced diabetes reduced the structural properties of the femoral neck (trabecular bone volume, trabecular thickness and collagen fiber content). The femoral neck mechanical properties (maximum load and tenacity) were also impaired in the diabetic rats. Insulin therapy partially reversed the damage induced by diabetes on the structural properties of the bone and mitigated the reductions in the mechanical properties of the bone. The combination of therapies further increased the femoral neck trabecular bone volume (∼30%), trabecular thickness (∼24%), collagen type I (∼19%) and type III (∼13%) fiber contents, maximum load (∼25%) and tenacity (∼14%).

**CONCLUSIONS:**

Eight weeks of swimming training potentiates the recovery of femoral neck strength in young rats with severe streptozotocin-induced diabetes under insulin therapy.

## INTRODUCTION

The risk of high hip fracture in people with type 1 diabetes mellitus (T1DM) is thought to be caused by poorer bone quality, which is related to impaired bone structure and tissue integrity 1,2,3. Long periods of hyperglycemia lead to nonenzymatic glycation 4 in bone tissue along with augmented cross-links in the bone collagen matrix, thus reducing bone formation 1,5 and increasing bone brittleness and fragility 6,7. Poor bone quality has been reported in adolescents with T1DM 8 and in a young rat model of streptozotocin (STZ)-induced diabetes; the characteristics of poor bone quality included impaired structure and mechanical properties of the femoral neck and impaired resistance to fracture 9,10. Fractures of the femoral neck account for over 50% of the hip fractures that occur in populations with elevated bone fragility (i.e., osteoporosis) 11.

Insulin therapy is essential for glycemic control and is linked with steady bone mineral density (BMD) as well as marked decreases (i.e., 38%) in bone resorption in patients with T1DM 12. In animal models, insulin reversed the reduction in BMD caused by STZ 13 and mitigated the deleterious effects of STZ on the structure and function of the femoral neck in young rats experimentally exposed to severe hyperglycemia 9.

Regular exercise is recommended to counteract bone fragility because of the mechanical stimulation and benefits to bone health that are associated with exercise 14. Therefore, swimming is an attractive therapeutic option for people with high bone fragility, such as those with severe T1DM, because of its safety, especially in relation to falls and excessive loads. Despite the lower impact of swimming exercise and the consequent osteogenic benefits compared to those from impact exercises 15, swimming has been shown to significantly improve femoral structural and mechanical properties in osteopenic rats 16,17.

Therefore, in this study, we employed a rat model of STZ-induced diabetes to test whether swimming training improves the strength of the femoral neck in young rats under insulin therapy.

## METHODS

### Animals

Forty-day-old male Wistar rats were allocated into one of the following six experimental groups (n=10 each group): control sedentary (CS), control exercise (CE), diabetic sedentary (DS), diabetic exercise (DE), diabetic sedentary + insulin (DSI), and exercise + insulin (DEI). The rats were housed in an environment with controlled humidity (60-70%), a controlled light-dark cycle (12/12 hours) and controlled temperature (∼22°C) and had free access to water and commercial chow. All experimental procedures were approved by the Ethics Committee on Animal Use of the Universidade Federal de Viçosa, Brazil (Protocol n. 51/ 2011), and the study was performed in accordance with international ethical standards 18.

### Induction of diabetes and blood glucose monitoring

After fasting for 12 hours, the animals from the diabetic groups received a single intraperitoneal injection (60 mg/kg of body weight (BW)) of STZ (Sigma-Aldrich, USA) diluted in 1.0 ml of a buffer solution (sodium citrate - 0.1 M, pH 4.5). The control rats were injected with the same dose of only the buffer. To confirm the induction of diabetes, one week after STZ injection, the blood glucose (BG) levels of the animals were measured (One Touch Ultra - Johnson & Johnson, Mexico) at rest after fasting for 12 hours. The rats that exhibited fasting hyperglycemia (i.e., BG levels ≥300 mg\dL) were considered diabetic. BG was monitored weekly on Monday mornings at 68 and 15 hours after the training sessions and insulin injections performed on Friday, respectively.

### Swimming training and insulin therapy

After 7 days of hyperglycemia, the animals in the DE, DEI and CE groups started the training program in which they swam 5 days/wk (Monday to Friday) for 8 weeks. This training program was adapted from a program discussed in a previous study 19. Briefly, in the 1^st^ week, the rats swam for 10 min/day, and the exercise duration was then increased by 10 min/day with no additional load (Table 1). By the 2^nd^ week, the duration of the exercise was increased again by 10 min/day until it reached 90 min with no additional load. Such conditions were maintained during the 3^rd^ week. From the 4^th^ week on, the swimming duration of 90 min/day was maintained, and a progressive load of 1% of BW was added to the tails of the animals weekly until the load reached 5% on the 8^th^ week (Table 1).

The rats from the DSI and DEI groups received human insulin at a dose of 2-4 U/day/rat (i.e., 1U per 60 g of BW) for the same 8-week period. The insulin therapy started with a daily dose of 2 units (subcutaneous) at 6 p.m., 6 hours after the training session. To simulate severe T1DM, the insulin dosage was adjusted gradually to maintain an average BG level of ∼300 mg/dL.

### Sample collection

Forty-eight hours after the end of the 8-week swimming program, the animals were euthanized and their right and left femurs were removed and dissected from the connective tissue. The left femur was fixed in neutral buffered formalin (10%) and used for histomorphometry. The right femur was immersed in saline solution and stored at -20°C until its use in the femoral neck mechanical testing.

### Femoral neck histomorphometry

**Figure 1:**
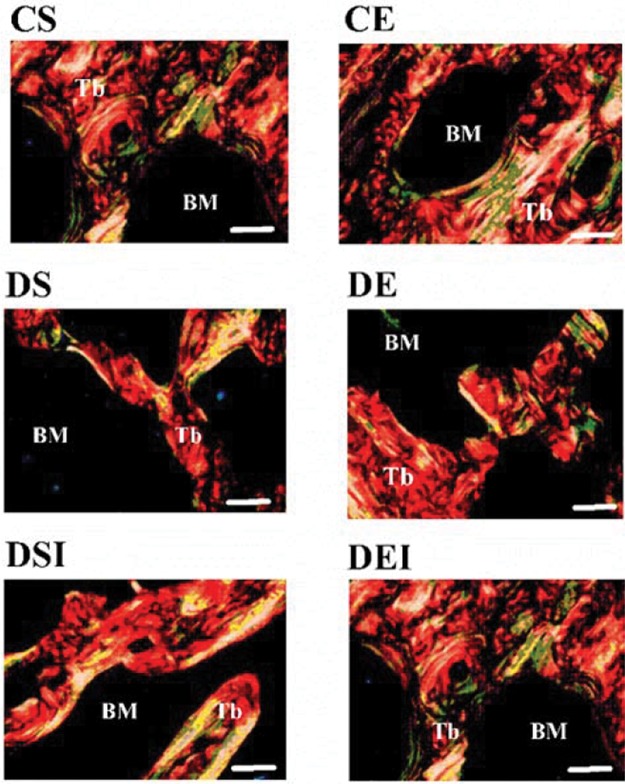
Representative photomicrographs of the femoral necks of the animals in the experimental groups. Tb, trabeculae. BM, bone marrow. Red and yellow colors, collagen type I fiber. Green color, collagen type III fiber. Sirius Red staining. Magnification, 200x. White bar, 100 μm. CS: control sedentary; CE: control exercise; DS: diabetic sedentary; DE: diabetic exercise; DSI: diabetic sedentary plus insulin; DEI: diabetic exercise plus insulin.

The left femur was decalcified and dehydrated, and longitudinal sections (5 µ-thick) were cut from the midneck region using a microtome (Leica 2065, Germany) and were affixed to histology slides as described previously 10. Slides stained with hematoxylin and eosin (H&E) were used to determine the trabecular bone volume per total bone volume (BV/TV), while the slides stained with Sirius Red were used to assess the collagen content. Representative photomicrographs of femoral neck collagen type I (red and yellow colors) and type III (green color) as well as the trabeculae and bone marrow of the animals in the experimental groups are shown in Figure 1.

The BV/TV was measured as described previously 10. Briefly, 5 images (200x) in distinct fields from each rat were digitalized using a photomicroscope (Olympus Biological CX31, Japan) equipped with the analySIS^®^ getIT software (Olympus Soft Imaging Solutions GmbH). The images were analyzed using Image-Pro Plus software, version 4.5.0.29 (Media Cybernetics, USA). The percentage of trabecular bone calculated from the images was used to express BV/TV. The percentage of bone marrow calculated from the images was used to express trabecular spacing. Trabecular thickness was determined by measuring the width of all trabeculae in the images (5 images per rat). Because the diabetic rats exhibited reduced BV/TV, we used the 5 thinnest trabeculae of each rat for comparison purposes.

The collagen content was assessed as described previously 20. In brief, we digitalized 5 images (200x) in distinct fields from each rat using a microscope (Olympus AX-70) with polarized light equipped with SPOT Basic Software, version 3.5.9 (Diagnostic Instruments, USA). The images were analyzed using Image-Pro Plus software (version 4.5.0.29 - Media Cybernetics). The collagen type I and type III fibers were counted and expressed as percentages.

### Measurements of femoral neck mechanical properties

The mechanical properties of the femoral neck were measured in the right femurs as described previously 10. In brief, to measure the strength of the femoral neck, we placed the femur axially in a supportive holder mounted on a computer-controlled universal testing machine (EMIC, DL 3000, Brazil) equipped with a 2000-N load cell. The loading force was applied downward to the top of the femoral head at a constant speed (0.5 mm/min) using a loading cup until the femoral neck fractured. Data were recorded at a frequency of 60 Hz and converted into a load-displacement curve from which we determined the maximum load, stiffness, tenacity, yield point energy and postyield energy. Since diabetes reduced the BW of the rats, the femoral neck mechanical properties were normalized by BW.

### Statistical analysis

A two-way repeated-measures analysis of variance (ANOVA) and Tukey's post hoc test were used to compare the BW and BG data. To compare the structural and mechanical data, we used the two-way ANOVA followed by Tukey's post hoc test.

## RESULTS

### Blood glucose and body weight

**Figure 2:**
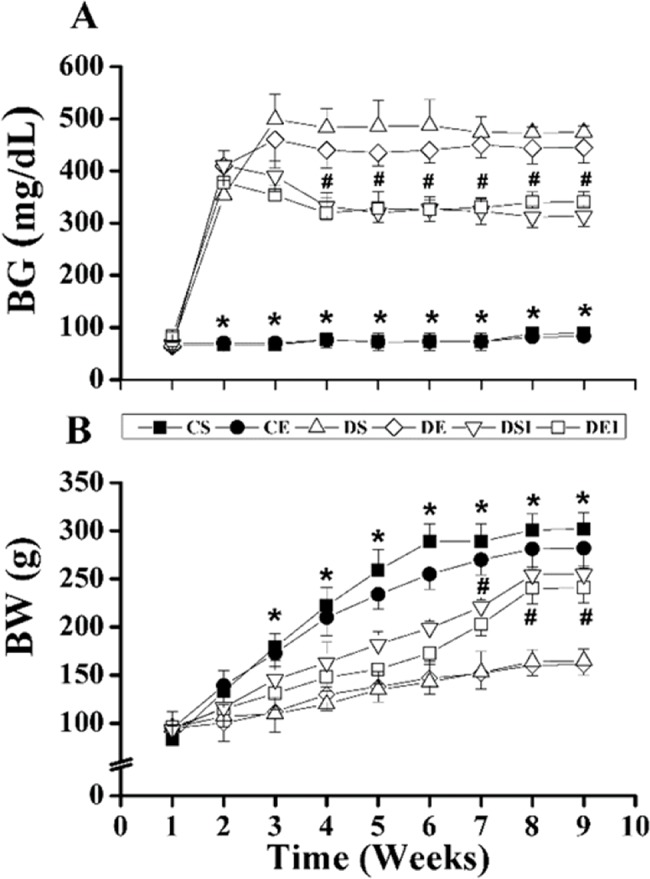
(A) Blood glucose (BG) and (B) body weight (BW) changes over the experimental period. The STZ injection was given at the start of week 1, and the swimming training plus insulin therapy began at the start of week 2. Data are expressed as the means ± SEMs of 10 rats in each group. CS: control sedentary; CE: control exercise; DS: diabetic sedentary; DE: diabetic exercise; DSI: diabetic sedentary plus insulin; DEI: diabetic exercise plus insulin. *^#^Significant difference between groups, *p*<0.05; *vs. DS, DE, DSI and DEI, ^#^*vs*. DS, DE, CS and CE.

Diabetes augmented BG levels to ∼500 mg/dL from week 2 until week 8 (Figure 2A). Insulin treatment reduced the BG levels to ∼320 mg/dL in both the sedentary and trained diabetic rats from week 4. Exercise training did not affect BG levels in the control or diabetic rats, and the combination of treatments had no effect on BG levels. Independent of the treatments, the control rats presented a higher BW than the diabetic rats from week 3 onward (Figure 2B). Insulin therapy partially restored the BWs of the diabetic rats, and the difference in BW between the diabetic insulin-treated rats and nontreated rats was significant from week 7 on. Exercise training had no effect on BW in the control or diabetic rats, and the combination of treatments did not affect BW.

### Femoral neck structural properties

**Figure 3:**
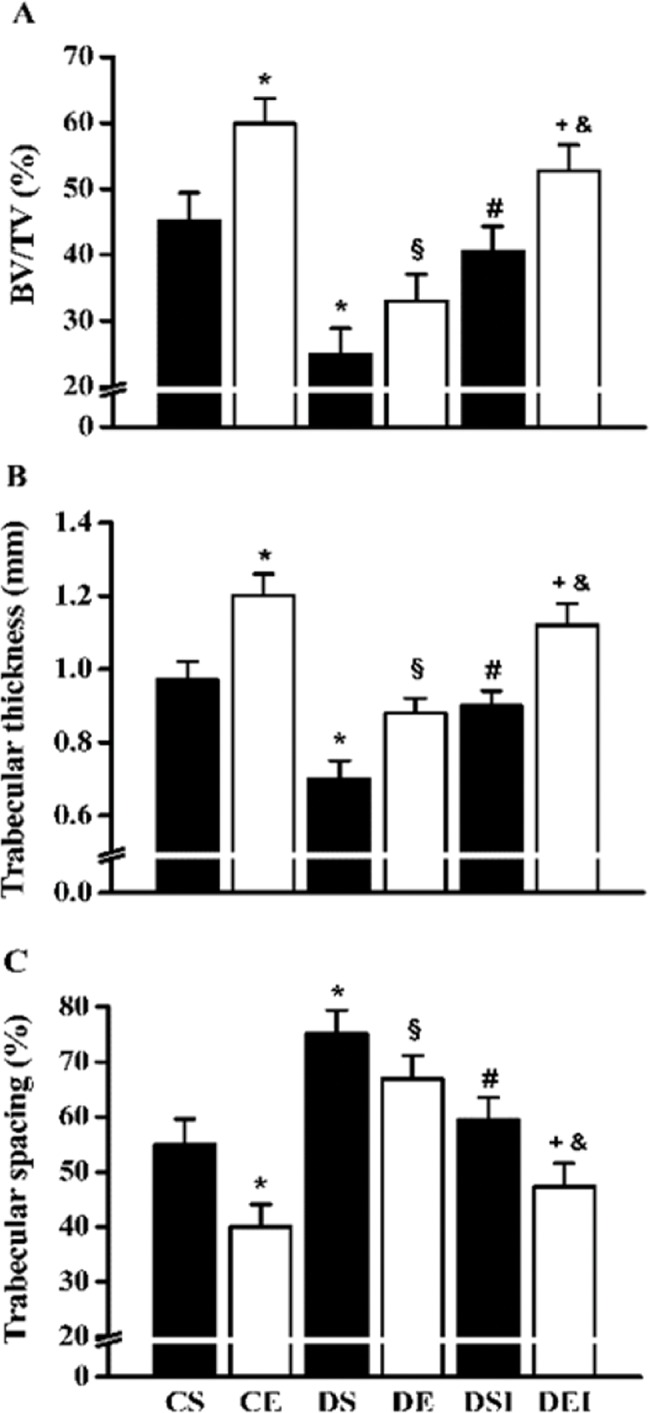
Femoral neck trabecular bone volume (BV/TV) (A), trabecular thickness (B) and trabecular spacing (C). Data are expressed as the means ± SEMs of 5 images per rat of the 10 rats in each group. CS: control sedentary; CE: control exercise; DS: diabetic sedentary; DE: diabetic exercise; DSI: diabetic sedentary plus insulin; DEI: diabetic exercise plus insulin. *^§^^#+&^Significant difference between groups, *p*<0.05; *vs. CS, ^§^*vs*. CE, ^#^*vs*. DS, ^+^*vs*. DE, ^&^*vs*. DSI.

The BV/TV was diminished in the diabetic rats (∼45%; Figure 3A). However, insulin therapy restored the BV/TV to the levels of the control rats (∼62% increase). The swimming program increased the BV/TV in control rats (∼33%), but in the diabetic rats that were not treated with insulin, this effect was not statistically significant. Nevertheless, the combination of therapies further increased the femoral neck BV/TV (∼30%). Diabetes reduced (∼28%) and increased (∼37%) the trabecular thickness (Figure 3B) and spacing (Figure 3C), respectively. In contrast, insulin therapy increased (∼28%) and reduced (∼21%) the trabecular thickness and spacing, respectively. The swimming training program increased the trabecular thickness (∼24%) and diminished the trabecular spacing (∼27%) in the femoral neck of the control rats. However, in the diabetic rats that were not treated with insulin, the effects of exercise on these parameters were not significantly different. When the therapies were combined, the trabecular thickness and spacing in the diabetic rats were further increased (∼24%) and reduced (∼21%), respectively.

**Figure 4:**
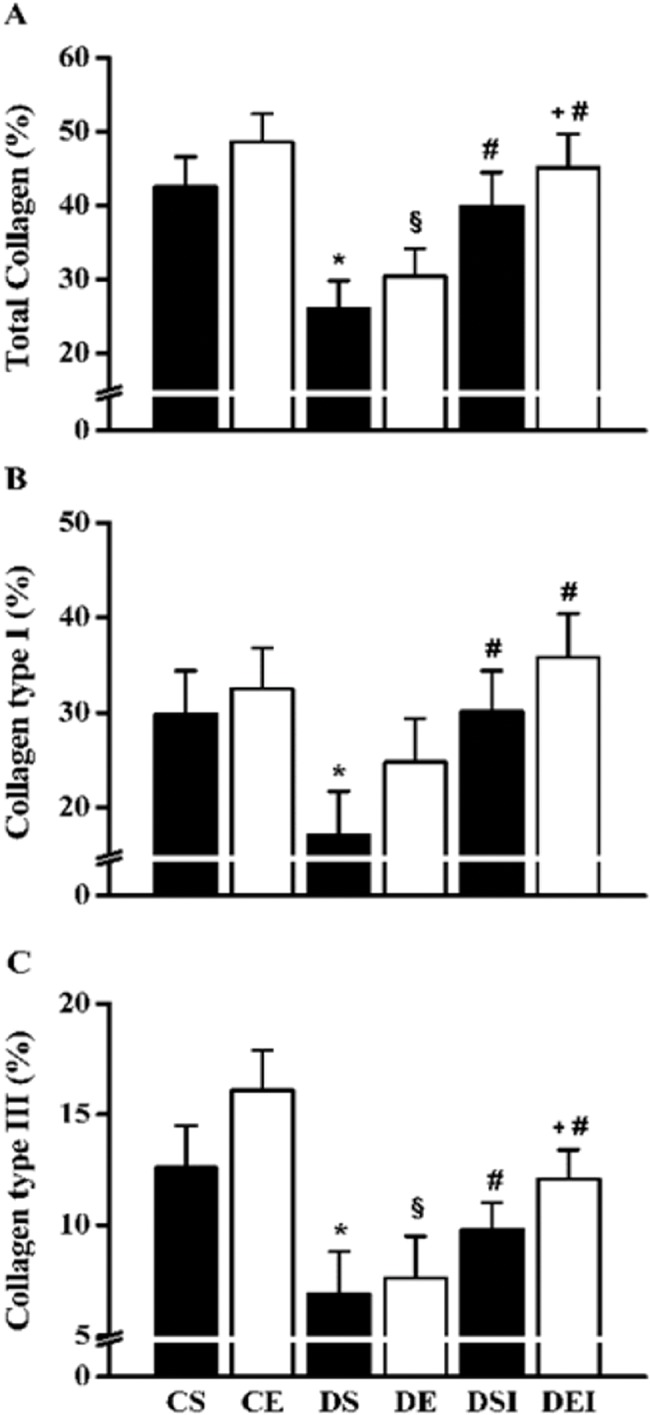
Femoral neck total collagen (A), collagen type I (B) and type III (C) fibers. Data are expressed as the means ± SEMs of 5 images per rat of the 10 rats in each group. CS: control sedentary; CE: control exercise; DS: diabetic sedentary; DE: diabetic exercise; DSI: diabetic sedentary plus insulin; DEI: diabetic exercise plus insulin. *^§^^#+&^Significant difference between groups, *p*<0.05; *vs. CS, ^§^*vs*. CE, ^#^*v*s. DS, ^+^*vs*. DE, ^&^*vs*. DSI.

Diabetes reduced the total collagen content (∼39%), which was partially restored by insulin therapy (∼53% increase; Figure 4 A) and completely restored when swimming training was combined with insulin therapy (∼13% increase). Collagen type I was diminished in the diabetic rats (∼43%) (Figure 4 B); however, it was almost completely restored by insulin therapy alone (∼76% increase), and the level was further increased by the combination of insulin and exercise training (∼19%). Collagen type III was also diminished in the diabetic rats (∼45%) (Figure 4 C); nevertheless, it was partially restored by insulin therapy alone (∼40% increase), and the level was further increased by the combination of insulin therapy and swimming training (∼23%). Swimming training alone did not significantly affect these collagen parameters.

### Femoral neck mechanical properties

The maximum load was significantly reduced in the diabetic rats (∼35%), and it was partially restored by insulin therapy (∼28% increase) (Table 2). The exercise training increased the maximum load in the control rats (∼26%) and had no effect on the diabetic rats that were not receiving insulin therapy. The combination of treatments, however, further increased (∼25%) the femoral neck maximum load in the diabetic rats. Diabetes status did not affect femoral neck stiffness. Insulin therapy, either alone or in combination with swimming training, had no effect on bone stiffness. Likewise, no effect of swimming training alone was observed. The femoral neck tenacity was significantly reduced in the diabetic rats (∼43%) and was partially restored by insulin therapy (∼47% increase). The femoral neck tenacity was further increased by the combination of insulin and exercise training (∼14%). Swimming training alone increased the femoral neck tenacity (∼35%) in only the control rats. The yield point energy was not significantly reduced in the diabetic rats. However, insulin therapy alone enhanced the yield point energy (∼56%) and further increased this parameter when combined with swimming training (∼13%). Swimming training alone increased the yield point energy in only the control rats (∼16%). The postyield energy was not significantly different among the groups and was not significantly affected by diabetes status, insulin therapy or swimming training.

## DISCUSSION

In this study, severe diabetes markedly reduced the BV/TV in the femoral neck and subsequently resulted in negative changes in trabecular thickness (i.e., diminished) and spacing (i.e., augmented). We also observed reduced collagen fibers in the diabetic rats. The diminished secretion and/or action of insulin, which is associated with the formation of advanced glycation end-products (AGEs) induced by oxidative stress and hyperglycemia, debilitates new bone formation by decreasing osteoblast activity and increasing osteoclast activity, thus reducing bone mass 21,22. In a rat model of diabetes, active osteoblasts may revert into inactive bone-lining cells, and a reduction in the proliferation of preosteoblastic cells has been proposed 23. The formation of AGEs also inhibits type I collagen synthesis 24 and the attachment of osteoblasts to the collagen matrix 22. Thus, since the collagen type III network plays a pivotal role in bone strength as it facilitates musculoskeletal exchange 25, the reduction in collagen content found in the femoral neck of the diabetic rats also contributed to the reduced trabecular BV/TV and impaired mechanical properties (see below).

Insulin therapy restored the femoral neck BV/TV in the diabetic rats, which was also reflected in the recovery of trabecular thickness and spacing. Likewise, insulin therapy restored the collagen content in the femoral neck of the diabetic rats. Insulin exerts an anabolic effect on bone and regulates bone turnover 26. Regarding collagen fibers, insulin acts directly on osteoblasts to increase bone collagen synthesis 27. Indirectly, by reducing BG levels, insulin decreases nonenzymatic glycation and, hence, the formation and accumulation of AGEs; insulin also decreases the formation of cross-links in the bone collagen matrix, thereby reducing its consequent mineralization 4.

While swimming training alone did not counteract the severe damage associated with T1DM, our results showed that combining insulin therapy with swimming training for 8 weeks further improved the recovery of the BV/TV and collagen fiber content (i.e., a major increase in type III collagen fibers) in the femoral neck of diabetic rats. This finding indicates that swimming training provided supplementary osteogenic stimuli in addition to the effects of insulin. Nonweight-bearing exercise, such as swimming, positively influences the skeleton indirectly by promoting increases in muscle mass and strength, which stimulates mechanoreceptors to trigger biological signals in the bone 12 and alter hormonal levels, leading to bone deposition 28. The collagen content in the femoral neck of nondiabetic rats has also been shown to be increased by swimming training 10. It has been suggested that the collagen fibers of the femoral neck at the point of muscle insertion are augmented as a result of forces exerted by the strong musculature 29.

Along with worsened structural properties, the maximum load and tenacity of the femoral neck were impaired by severe diabetes. The maximum load is a measure of bone strength that is underpinned by bone mass and collagen. Therefore, the reduced femoral neck BV/TV and collagen content resulted in decreased trabecular bone resistance to fracture in the diabetic rats. The lower tenacity that was observed in the T1DM rats is in line with the reduced femoral neck collagen content in these rats, as bone tenacity is determined primarily by the unmineralized matrix 30. In fact, reduced bone collagen content is suggested to result in bone loss and diminished bone strength in T1DM 4, resulting in fragile bones. Moreover, the increased cross-links in the bone collagen matrix lead to premature increases in bone brittleness 6,7. However, other factors, such as bone microarchitecture and microdamage accumulation, may negatively affect bone biomechanical integrity in diabetic rats 31.

Treatment with insulin improved both femoral neck maximum load and tenacity in diabetic rats. Although swimming training alone did not efficiently counterbalance the T1DM-associated deteriorations, the combination of swimming training and insulin therapy further improved the femoral neck strength, as an additional increase in the maximum load and tenacity was found in the diabetic rats. Such benefits in these mechanical properties were associated with restored femoral neck structural properties resulting from insulin and the combination of insulin and swimming exercise, as mentioned above. These findings reinforce the usefulness of swimming training as an osteogenic stimulus, especially in populations under treatment for bone fragility. Nevertheless, swimming training may improve bone strength by positively affecting other factors, such as trabecular bone microarchitecture, thus benefiting the structural efficiency of the trabecular bone 16,17.

Despite the differences in bone loading patterns between rats and humans, the effects of swimming training on diabetic rat trabecular bone may be of clinical relevance for humans because of the impaired femoral neck properties and high risk for fracture in patients with T1DM. Moreover, the results obtained here represent a scenario of severe T1DM (i.e., BG ≥300 gm/dL) and may not be generalizable to patients under insulin therapy, although a clinical study reported reduced bone mass in young subjects with moderate T1DM who had good metabolic control 32.

Finally, this study has limitations. First, serum and urinary bone biochemical markers were not assessed, and the bone mass in diabetic rats may have been influenced by urinary calcium and magnesium excretion, as well as intestinal calcium absorption 33. Second, insulin concentrations were not assessed. Nevertheless, the measurement of BG levels is a good indicator of insulin deficiency.

In conclusion, these results indicate that eight weeks of swimming training potentiates the recovery of femoral neck strength in young rats with severe STZ-induced diabetes under insulin therapy.

## AUTHOR CONTRIBUTIONS

Gomes GJ was responsible for the intellectual and scientific content of the study, the technical procedures that were followed, and the acquisition and interpretation of the data. Del Carlo RJ was responsible for the intellectual and scientific content of the study and the critical revision of the manuscript. da Silva MF, Cunha DNQ, da Silva E and Silva KA were responsible for the acquisition and interpretation of the data and the technical procedures that were followed. Carneiro-Junior MA and Primola-Gomes TN were responsible for the intellectual and scientific content of the study and the interpretation of the data. Natali AJ was responsible for the design and intellectual and scientific content of the study, for writing and critically revising the manuscript and for securing funding. All authors approved the final version of the manuscript.

## Figures and Tables

**Table 1:** Swimming training schedule.

Week	Load (% BW)	Monday (Time - min)	Tuesday (Time - min)	Wednesday (Time - min)	Thursday (Time - min)	Friday (Time - min)
1	0	10	20	30	40	50
2	0	60	70	80	90	90
3	0	90	90	90	90	90
4	1	90	90	90	90	90
5	2	90	90	90	90	90
6	3	90	90	90	90	90
7	4	90	90	90	90	90
8	5	90	90	90	90	90

BW: body weight.

**Table 2:** Femoral neck mechanical properties.

	CS	CE	DS	DE	DSI	DEI
Maximum load (N)	0.391±0.031	0.492±0.029^*^	0.252±0.030^*^	0.281±0.029^§^	0.323±0.030^#^	0.404±0.031^#&^
Stiffness (N/mm)	0.441±0.060	0.611±0.089^*^	0.510±0.061	0.532±0.041	0.382±0.051	0.471±0.039
Tenacity (mJ)	0.260±0.030	0.352±0.031^*^	0.150±0.030^*^	0.181±0.029^§^	0.225±0.028^#^	0.252±0.030^#§^
Yield point energy (mJ)	0.057±0.009	0.087±0.008^*^	0.048±0.009	0.052±0.009^§^	0.075±0.008^#^	0.085±0.008^#+^
Postyield energy (mJ)	0.239±0.043	0.297±0.040	0.201±0.043	0.224±0.040	0.180±0.043	0.185±0.040

Data are expressed as the means ± SEMs of 10 animals in each group. CS: control sedentary; CE: control exercise; DS: diabetic sedentary; DE: diabetic exercise; DSI: diabetic sedentary plus insulin; DEI: diabetic exercise plus insulin. ^*§#+&^ Significant difference between groups, *p*<0.05; ^*^vs. CS, ^§^*vs*. CE, ^#^*vs*. DS, ^+^*vs*. DE, ^&^*vs*. DSI.

## References

[1] Napoli N, Chandran M, Pierroz DD, Abrahamsen B, Schwartz AV, Ferrari SL (2017). Mechanisms of diabetes mellitus-induced bone fragility. Nat Rev Endocrinol.

[2] Sellmeyer DE, Civitelli R, Hofbauer LC, Khosla S, Lecka-Czernik B, Schwartz AV (2016). Skeletal metabolism, fracture risk, and fracture outcomes in type 1 and type 2 diabetes. Diabetes.

[3] Vestergaard P, Rejnmark L, Mosekilde L (2009). Diabetes and its complications and their relationship with risk of fractures in type 1 and 2 diabetes. Calcif Tissue Int.

[4] Saito M, Fujii K, Mori Y, Marumo K (2006). Role of collagen enzymatic and glycation induced cross-links as a determinant of bone quality in spontaneously diabetic WBN/Kob rats. Osteoporos Int.

[5] Saito M, Marumo K (2010). Collagen cross-links as a determinant of bone quality: a possible explanation for bone fragility in aging, osteoporosis, and diabetes mellitus. Osteoporos Int.

[6] Nyman JS, Even JL, Jo C, Herbert EG, Murry MR, Cockrell GE (2011). Increasing duration of type 1 diabetes perturbs the strength-structure relationship and increases brittleness of bone. Bone.

[7] Saito M, Fujii K, Soshi S, Tanaka T (2006). Reductions in degree of mineralization and enzymatic collagen cross-links and increases in glycation-induced pentosidine in the femoral neck cortex in cases of femoral neck fracture. Osteoporos Int.

[8] Soto N, Pruzzo R, Eyzaguirre F, Iãiguez G, López P, Mohr J (2011). Bone mass and sex steroids in postmenarcheal adolescents and adult women with Type 1 diabetes mellitus. J Diabetes Complications.

[9] Hou JC, Zernicke RF, Barnard RJ (1993). Effects of severe diabetes and insulin on the femoral neck of the immature rat. J Orthop Res.

[10] Silva KA, Del Carlo RJ, Matta SLP, Louzada MJQ, Rodrigues AC, Silva MF (2014). Effects of swimming training on the femoral neck strength in growing rats with untreated streptozotocin-induced diabetes. Int SportMed J.

[11] Thorngren KG, Hommel A, Norrman PO, Thorngren J, Wingstrand H (2002). Epidemiology of femoral neck fractures. Injury.

[12] Hamrick MW, Samaddar T, Pennington C, McCormick J (2006). Increased muscle mass with myostatin deficiency improves gains in bone strength with exercise. J Bone Miner Res.

[13] Erdal N, Gurgul S, Demirel C, Yildiz A (2012). The effect of insulin therapy on biomechanical deterioration of bone in streptozotocin (STZ)-induced type 1 diabetes mellitus in rats. Diabetes Res Clin Pract.

[14] Beck BR, Daly RM, Singh MA, Taaffe DR (2017). Exercise and Sports Science Australia (ESSA) position statement on exercise prescription for the prevention and management of osteoporosis. J Sci Med Sport.

[15] Gómez-Bruton A, Gónzalez-Aguero A, Gómez-Cabello A, Casajús JA, Vicente-Rodríguez G (2013). Is bone tissue really affected by swimming? A systematic review. PloS One.

[16] Ju YI, Sone T, Ohnaru K, Tanaka K, Fukunaga M (2015). Effect of swimming exercise on three-dimensional trabecular bone microarchitecture in ovariectomized rats. J Appl Physiol.

[17] Kang YS, Kim SH, Kim JC (2017). Effects of swimming exercise on high-fat diet-induced low bone mineral density and trabecular bone microstructure in rats. J Exerc Nutrition Biochem.

[18] Harriss DJ, Macsween A, Atkinson G (2017). Standards for Ethics in Sport and Exercise Science Research: 2018 Update. Int J Sports Med.

[19] Thrailkill KM, Lumpkin CK, Bunn RC, Kemp SF, Fowlkes JL (2005). Is insulin an anabolic agent in bone? Dissecting the diabetic bone for clues. Am J Physiol Endocrinol Metab.

[20] Drummond LR, Del Carlo RJ, Melo SFS, Carneiro-Júnior MA, Silva KA, Rodrigues AC (2013). Enhanced femoral neck strength in response to weightlifting exercise training in maturing male rats. Int SportMed J.

[21] Duarte VM, Ramos AM, Rezende LA, Macedo UB, Brandão-Neto J, Almeida MG (2005). Osteopenia: a bone disorder associated with diabetes mellitus. J Bone Miner Metab.

[22] McCarthy AD, Uemura T, Etcheverry SB, Cortizo AM (2004). Advanced glycation endproducts interefere with integrin-mediated osteoblastic attachment to a type-I collagen matrix. Int J Biochem Cell Biol.

[23] Weiss RE, Reddi AH (1980). Influence of experimental diabetes and insulin on matrix-induced cartilage and bone differentiation. Am J Physiol.

[24] Katayama Y, Akatsu T, Yamamoto M, Kugai N, Nagata N (1996). Role of nonenzymatic glycosylation of type I collagen in diabetic osteopenia. J Bone Miner Res.

[25] Luther F, Saino H, Carter DH, Aaron JE (2003). Evidence for an extensive collagen type III/VI proximal domain in the rat femur. I. Diminution with ovariectomy. Bone.

[26] Yang J, Zhang X, Wang W, Liu J (2010). Insulin stimulates osteoblast proliferation and differentiation through ERK and PI3K in MG-63 cells. Cell Biochem Funct.

[27] Kream BE, Smith MD, Canalis E, Raisz LG (1985). Characterization of the effect of insulin on collagen synthesis in fetal rat bone. Endocrinology.

[28] Maïmoun L, Sultan C (2009). Effect of physical activity on calcium homeostasis and calciotropic hormones: a review. Calcif Tissue Int.

[29] Saino H, Luther F, Carter DH, Natali AJ, Turner DL, Shahtaheri SM (2003). Evidence for an extensive collagen type III proximal domain in the rat femur. II. Expansion with exercise. Bone.

[30] Boivin G, Bala Y, Doublier A, Farlay D, Ste-Marie LG, Meunier PJ (2008). The role of mineralization and organic matrix in the microhardness of bone tissue from controls and osteoporotic patients. Bone.

[31] Kohn DH, Sahar ND, Wallace JM, Golcuk K, Morris MD (2009). Exercise alters mineral and matrix composition in the absence of adding new bone. Cells Tissues Organs.

[32] Moyer-Mileur LJ, Slater H, Jordan KC, Murray MA (2008). IGF-1 and IGF-binding proteins and bone mass, geometry, and strength: relation to metabolic control in adolescent girls with type 1 diabetes. J Bone Miner Res.

[33] Blakytny R, Spraul M, Jude EB (2011). Review: The diabetic bone: a cellular and molecular perspective. Int J Low Extrem Wounds.

